# Supplementary material for: Probing the effects of nonannular lipid binding on the stability of the calcium pump SERCA
Source: Sci Rep. 2019 Mar 4;9:3349. doi: 10.1038/s41598-019-40004-y (PMC6399444; doi:10.1038/s41598-019-40004-y)
Supplement: Supplementary file 1 — Supplementary Information [file 41598_2019_40004_MOESM1_ESM.pdf]

Supplementary Information

Probing the effects of nonannular lipid binding on the stability of the calcium pump SERCA

L. Michel Espinoza-Fonseca

Center for Arrhythmia Research, Department of Internal Medicine, Division of Cardiovascular Medicine, University of Michigan, Ann Arbor, MI 48109, USA

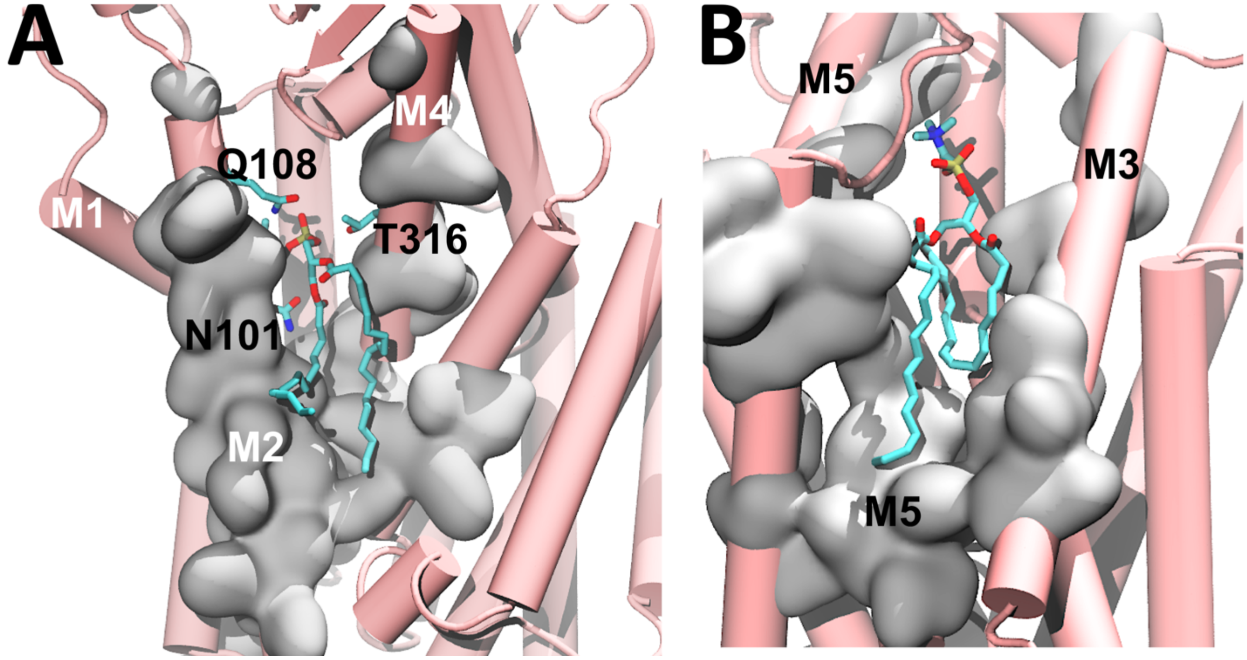

Figure S1. Binding of nonannular lipids to SERCA during the early stages of the E2 to E1 transition of the pump. (A) Upon deprotonation of transport site residues Glu309 and Glu771 and in the absence of an initially bound nonannular lipid, the pump undergoes structural changes in the transmembrane and cytosolic domains that initiate the transition toward the competent E1 state[1,2]. In a previous study by our group[2], we found that during these structural change, a single lipid molecule binds to the nonannular site at t=0.2 μs and remains bound to the pump for the reminder of the simulation time (t=3.6 μs). Remarkably, the nonannular lipid interacts with residues that participate in nonannular lipid binding to this site, i.e., Asn101, Gln108 and Thr316. (B) In the same trajectory of the early stages of the E2-to-E1 transition, metal-proton exchange at the transport sites induce a physical separation of helices M3 and M5, thus exposing several hydrophobic residues at the M3-M5 interface, which is occupied by a nonannular lipid. In both cases, these nonannular lipids appear to protect SERCA by 'sealing' transient hydrophobic patches in the transmembrane domain that result from the large structural changes in the pump during the E2-to-E1 transition. Based on these observations, we propose nonannular lipid binding is not necessary for the stability of the E2 state, but we speculate that it becomes functionally significant during the E2-to-E1 transition of the pump.

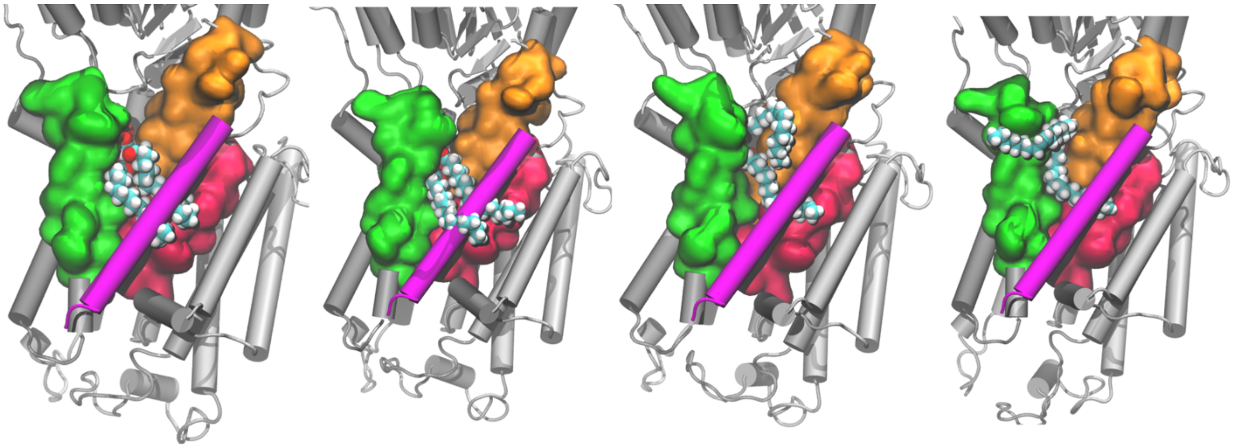

Figure S2. Location of the nonannular lipid tails within the canonical binding for SERCA regulatory proteins. We created several structural models of the interaction between the E2 state of SERCA (gray) and the transmembrane domain of phospholamban/sarcolipin (magenta). This structural model indicates that the lipid tails of the nonannular lipid (van der Waals spheres) protrude out of the nonannular site toward transmembrane helices M2 (green), M4 (orange) and M6 (red). These structural models suggest that the nonannular lipid competes with phospholamban and sarcolipin, thus inhibiting the formation of the complex between the E2 state and these regulatory proteins. This hypothesis is partially supported by x-ray crystallography studies showing that the absence of sarcolipin in the canonical regulatory site of SERCA[3]. Based on these structural models, we hypothesize that lipid binding to this site blocks the interaction between the E2 state of SERCA and its endogenous regulatory proteins.

References

1 Inesi, G., Lewis, D., Toyoshima, C., Hirata, A. & de Meis, L. Conformational fluctuations of the Ca2+-ATPase in the native membrane environment. Effects of pH, temperature, catalytic substrates, and thapsigargin. *J Biol Chem* **283**, 1189-1196, doi:M707189200 [pii]10.1074/jbc.M707189200 (2008).

2 Fernandez-de Gortari, E. & Espinoza-Fonseca, L. M. Preexisting domain motions underlie protonation-dependent structural transitions of the P-type Ca(2+)-ATPase. *Phys Chem Chem Phys* **19**, 10153-10162, doi:10.1039/c7cp00243b (2017).

3 Toyoshima, C. *et al.* Crystal structures of the calcium pump and sarcolipin in the Mg2+-bound E1 state. *Nature* **495**, 260-264, doi:10.1038/nature11899 [pii] (2013).
